# Differential involvement of Wnt signaling in Bmp regulation of cancellous versus periosteal bone growth

**DOI:** 10.1038/boneres.2017.16

**Published:** 2017-06-06

**Authors:** Guangxu He, Yu Shi, Joohyun Lim, Teresita Bellido, Jiangdong Ni, Fanxin Long

**Affiliations:** 1 Department of Orthopedics, The Second Xiangya Hospital, Central South University, Hunan, China; 2 Department of Orthopaedic Surgery, Washington University School of Medicine, St Louis, MO, USA; 3 Department of Anatomy and Cell Biology, Indiana University School of Medicine, Indianapolis, IN, USA; 4 Department of Developmental Biology, Washington University School of Medicine, St Louis, MO, USA

## Abstract

Bone morphogenetic proteins (Bmp) are well-known to induce bone formation following chondrogenesis, but the direct role of Bmp signaling in the osteoblast lineage is not completely understood. We have recently shown that deletion of the receptor Bmpr1a in the osteoblast lineage with *Dmp1-Cre* reduces osteoblast activity in general but stimulates proliferation of preosteoblasts specifically in the cancellous bone region, resulting in diminished periosteal bone growth juxtaposed with excessive cancellous bone formation. Because expression of sclerostin (SOST), a secreted Wnt antagonist, is notably reduced in the Bmpr1a-deficient osteocytes, we have genetically tested the hypothesis that increased Wnt signaling might mediate the increase in cancellous bone formation in response to Bmpr1a deletion. Forced expression of human SOST from a Dmp1 promoter fragment partially rescues preosteoblast hyperproliferation and cancellous bone overgrowth in the Bmpr1a mutant mice, demonstrating functional interaction between Bmp and Wnt signaling in the cancellous bone compartment. To test whether increased Wnt signaling can compensate for the defect in periosteal growth caused by Bmpr1a deletion, we have generated compound mutants harboring a hyperactive mutation (A214V) in the Wnt receptor Lrp5. However, the mutant Lrp5 does not restore periosteal bone growth in the Bmpr1a-deficient mice. Thus, Bmp signaling restricts cancellous bone accrual partly through induction of SOST that limits preosteoblast proliferation, but promotes periosteal bone growth apparently independently of Wnt activation.

## Introduction

Originally discovered in bone, bone morphogenetic proteins (Bmp) play essential roles in both embryogenesis and postnatal tissue homeostasis in mammals.^1,2,3^ Bmp proteins signal through the serine/threonine kinase receptors including four type I receptors (Bmpr1a, Bmpr1b, Acvrl1, Acvr1) and three type II receptors (Bmpr2, Acvr2a and Acvr2b).^4^ Binding of dimeric BMP proteins to a hetero-tetramer including two molecules of each receptor type leads to phosphorylation and activation of the type I receptor by the type II receptor with constitutively active kinase activity.^5^ In the best characterized mechanism, the type I receptors phosphorylate receptor Smads (Smad 1, 5, and 8) which in turn recruit the common partner Smad4 and other nuclear factors to regulate gene expression.^3,6,7^ In alternative pathways, Bmp proteins have been shown to activate TAK1-p38 and PI3K-Akt signaling axis.^4,6,8,9^ We have recently provided evidence that Bmpr1a signaling activates mTORC1 to regulate bone formation.^10^ Depending on the cellular context, Bmp may employ different effectors to control various biological processes.

Mouse knockout studies have established the essential role of Bmp in cartilage development. Deletion of Smad4 or a combination of Bmp ligands in the prechondrogenic mesenchyme has established that a threshold level of Bmp signaling via Smad4 is essential for chondrogenesis.^11,12,13^ In addition, deletion of Bmpr1a and Bmpr1b, or Smad1 and 5 in chondrocytes causes severe chondrodysplasia.^14,15^ Thus, Bmp signaling critically regulates multiple steps of cartilage development.

Mouse genetic studies have also revealed the importance of Bmp signaling in the osteoblast lineage. Knockout of Bmp2 in the limb mesenchyme (*Prx1-Cre*) greatly diminishes the strength of long bones in postnatal mice resulting in spontaneous fractures.^16^ Deletion of Bmpr1a or Smad4 in mature osteoblasts (*Og2-Cre*) decreases cancellous bone mass in young mice due to reduced bone formation, but leads to more bone at an older age due to less bone resorption.^17,18^ Remarkably, deletion of Bmpr1a with either *Col1-Cre^ER^* or *Dmp1-Cre* markedly increases cancellous bone mass, whereas deletion with *Dmp1-Cre* also diminishes periosteal bone growth.^10, 19,20,21^ We have further shown that Bmpr1a deletion with *Dmp1-Cre* reduces osteoblast activity but stimulates preosteoblast proliferation within the cancellous bone region.^10^ On the other hand, others have reported that Bmpr1a deletion with either *Col1-Cre^ER^* or *Dmp1-Cre* increases Wnt signaling which in turn suppresses osteoclastogenesis.^19^ Whether changes in Wnt signaling contribute to the regulation of preosteoblast proliferation or osteoblast activity by Bmp has not been determined.

Here we test the role of Wnt signaling in mediating Bmpr1a function in osteoblast lineage cells. Forced expression of the human sclerostin (SOST, a secreted Wnt antagonist) from a *Dmp1* promoter fragment partially rescued hyperproliferation of preosteoblasts in the *Dmp1-Cre*; *Bmpr1a^f/f^* mice. In contrast, expression of either SOST or a hyperactive form of the Wnt co-receptor Lrp5 did not modify the reduced osteoblast activity caused by the loss of Bmpr1a. Thus, Bmp signaling regulates bone formation through both Wnt-dependent and -independent mechanisms.

## Materials and methods

### Mouse strains

*Dmp1-Cre*,^22^ *Bmpr1a^f/f^*,^23^ *Dmp1-SOST*^24^ and *Lrp5^A214V/+^* (ref. 25) mouse strains are as previously described. The mouse strains were maintained in a mixed genetic background of mostly C57BL6 and some 129. All analyses were performed on sex-matched littermates including both males and females at the age of 33 days (P33). All mice were housed in a specific pathogen-free (SPF) barrier facility managed by Washington University Department of Comparative Medicine. The animals were group housed with a 12-h light cycle (6:00–18:00) and fed standard chow (PicoLab mouse diet 20, product number 5058). The Animal Studies Committee at Washington University approved all mouse procedures used in this study.

### Morphological analyses of bones

X-ray radiography was performed with Faxitron X-ray system (Faxitron X-ray Corp, Buffalo Grove, IL, USA) for 20-second exposures at 25 kV. Micro-computed tomography (μCT 40, Scanco Medical AG, Wayne, PA, USA) was performed on the tibia or the femur. Both procedures were performed on post-mortem tissues. Quantification of the cancellous bone was assessed by measuring 100 μCT slices (1.6 mm) immediately below the growth plate, whereas the total metaphyseal bone mass was calculated by including both cortical and cancellous bone in those μCT slices, both with a threshold of 240. For cortical bone parameters, 50 μCT slices (0.8 mm) from locations as indicated in the text were analyzed, with a threshold of 260. Other key parameters for μCT scan acquisition are as follows: voxel size 10 μm^3^, X-ray tube potential 55 kVp, X-ray intensity 145 μA, integration time 300 ms.^26^

Hematoxylin and eosin (H&E) was performed on paraffin sections with the thickness of 6 μm, following overnight fixation with neutral buffered 10% formalin and decalcification with 14% EDTA (pH 7.4) for 2 weeks at room temperature with daily changes of solution. For dynamic histomorphometry of postnatal mice, calcein (Sigma) dissolved in water (pH 7.2–7.4 adjusted with NaOH) was injected at 7.5 mg·kg^−1^ body weight intraperitoneally at 7 and 2 days, respectively, prior to killing. Bones were fixed in 70% ethanol, embedded in methyl-methacrylate and sectioned at 10 μm. Histomorphometric parameters were acquired with Bioquant Osteo II from three sections per mouse and three mice for each genotype.

### *In vivo* assays

For serum CTX-I assays, serum was collected through retro-orbital bleeding from mice starved for 6 h, and analyzed with the RatLaps ELISA kit (Immunodiagnostic Systems, Ltd., Gaithersburg, MD, USA) according to manufacturer's instructions. To collect serum, blood was collected with heparinized micro-hematocrit capillary tubes (22–362–566, Fisher Scientific, Pittsburgh, PA, USA), transferred to BD Microtainer SST Tubes (365967, Becton, Dickinson and Company, Franklin Lakes, NJ, USA). The SST tubes containing blood samples were then inverted five times and let sit at room temperature for 30 min to allow clotting before centrifugation for 90 s.

EdU (Invitrogen, Carlsbad, CA, USA) dissolved in water was injected intraperitoneally at 10 μg·g^−1^ body weight at 4 h before collection. Frozen sections were subjected to immunostaining for Osx (ab22552, Abcam, Cambridge, MA, USA) and Alexa Fluor 647 conjugated goat anti-rabbit secondary antibody (A21246, Invitrogen), followed by a click reaction according to manufacturer’s instructions (Click-iT EdU Alexa Fluor 488 Imaging Kit, Invitrogen). A non-immune IgG (5415S, Cell Signaling Technology, Danvers, MA, USA) was used as negative control. Images were acquired with the Nikon C-1 confocal system.

### Western blots of bone proteins

For western blots of bone extracts, femurs and tibiae from P33 mice were cleanly dissected with the epiphysis removed. After removing the marrow by centrifugation, bones were cut into small pieces and rinsed three times with ice-cold PBS. Bone pieces were snap-frozen in liquid nitrogen, pulverized at 2 000 r·min^−1^ for 20 s using a Mikro-Dismembrator (Sartorius, Gottingen, Germany) and then lysed with RIPA buffer containing protease inhibitors (cOmplete, cat# 11836145001, Roche, Basel, Switzerland) and phosphatase inhibitors (PhosSTOP, cat# 04906845001, Roche). Western blots were performed as previously described and the signals detected with Clarity ECL Substrate (Bio-Rad, Hercules, CA, USA).^27^ Western images were captured with Chemidoc (Bio-Rad).

### Immunostaining of sclerostin

Immununohistochemistry of sclerostin was performed as follows. Long bone sections were deparaffinized, briefly incubated in 3% H_2_O_2_ in methanol and rinsed in deionized water. The sections were first blocked with 5% normal serum and then incubated in biotinylated sclerostin antibody (BAF1589, R&D Systems, Minneapolis, MN, USA) at 1:500 in blocking solution. Streptavidin-HRP antibody and DAB substrate kit (Life Technologies, Carlsbad, CA, USA) were used according to manufacturer’s instructions. For negative control, the primary antibody was omitted from the procedure. For immunofluorescence detection of both human and murine sclerostin, a polyclonal antibody (ab75914, Abcam), together with Alexa Fluor 647 conjugated goat anti-rabbit secondary antibody (A21246, Invitrogen), was used on frozen sections of the femur from P33 mice. A non-immune IgG (5415S, Cell Signaling Technology) was used as negative control. For preparation of frozen sections, dissected bones were fixed with 4% PFA overnight at room temperature and incubated in 14% EDTA for 3 days with daily change of solution. The bones were then put in 30% sucrose overnight at 4 °C for cryoprotection and embedded in optimal cutting temperature (OCT) (Tissue-Tek, Torrance, CA, USA). Sections of 10 μm in thickness were obtained with a Leica cryostat equipped with Cryojane (Leica, Buffalo Grove, IL, USA), and kept at −20 °C until use. Fluorescent images were captured with the Nikon C-1 confocal system.

### Statistics

Statistical significance was calculated with either Student’s *t*-test, one-way analysis of variance (GraphPad Prism, La Jolla, CA, USA) or two-way Factorial analysis of variance for independent samples (vassarstats.net) as indicated in figure legends.

## Results

### Forced expression of SOST ameliorates cancellous but not cortical bone phenotype in Bmpr1a-deficient mice

**Figure 1:**
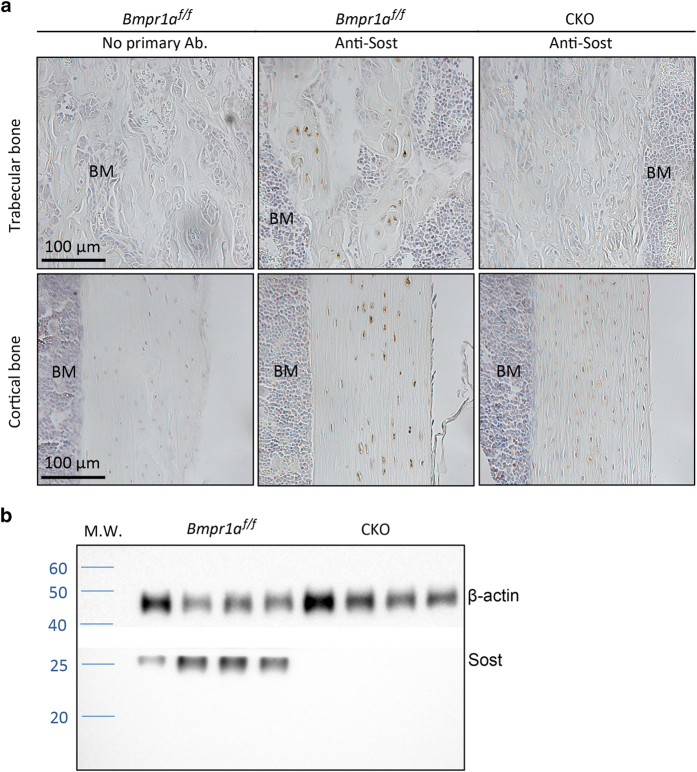
Deletion of Bmpr1a reduces SOST expression in osteocytes. (**a**) Representative images from immunohistochemistry of SOST on sections of the femur from littermate mice at P33. (**b**) Western blots with protein extracts from femurs and tibiae at P33. Each lane represents sample from a separate mouse. β-actin used as loading control. BM, bone marrow; CKO, *Dmp1-Cre*; *Bmpr1a^f/f^*; M.W., molecular weight markers; SOST, sclerostin.

As previous studies have implicated the regulation of SOST expression by Bmp, we examined the protein level of SOST in the bones of the *Dmp1-Cre*; *Bmpr1a^f/f^* (CKO) mice. Because we have previously analyzed the CKO mice at 33 days of age (P33), we conducted the current study at the same age to ensure consistency.^10^ The *Dmp1-Cre* transgene expresses Cre from a 9.6-kb Dmp1 promoter sequence, and the Bmpr1a^f^ allele has the second exon floxed and results in a complete loss of function when excised by Cre. Immunohistochemistry confirmed osteocytes as the predominant cell type expressing SOST in both cortical and cancellous bone of the control mice (*Bmpr1a^f/f^*) at P33 (Figure 1a, middle). In contrast, SOST was barely detectable in the same cell type of the CKO littermate (Figure 1a, right). Western blot analyses of protein extracts from the long bones corroborated the virtual absence of SOST in the CKO samples (Figure 1b). These results therefore confirm that SOST is markedly reduced in the Bmpr1a-deficient bones.

**Figure 2:**
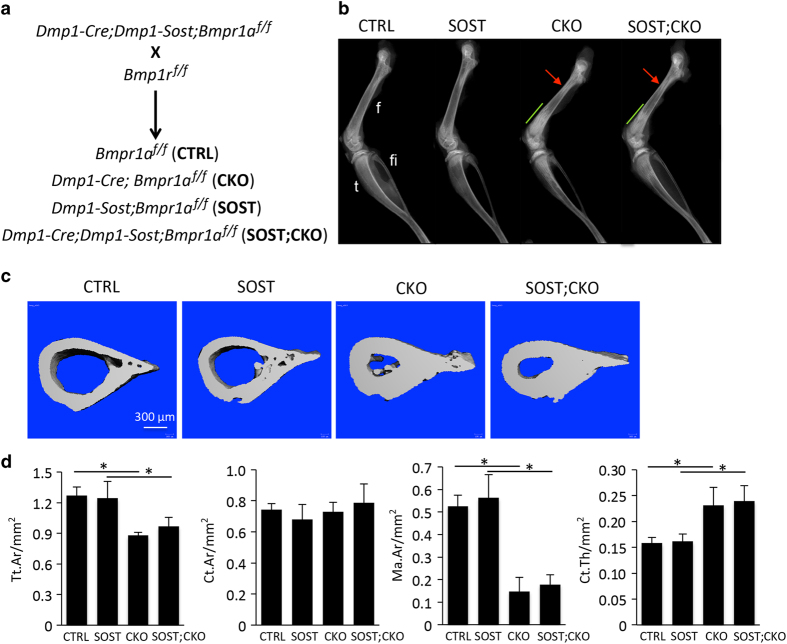
Forced expression of SOST does not modify bone diameters in Bmpr1a-deficient mice. (**a**) Mating scheme. (**b**) X-ray radiography of the hindlimb from littermate mice at P33. Arrows denote restricted region in the proximal femur specific to CKO and SOST;CKO mice. Lines indicate expanded cancellous bone region. (**c** and **d**) μCT images (**c**) or quantification (**d**) of cortical bone acquired at the regions marked by the arrows or the equivalent regions in (**b**). *P<0.001, one-way ANOVA, *n*=5 for CTRL and CKO, *n*=6 for SOST, *n*=7 for SOST;CKO. f, femur; fi, fibula; SOST, sclerostin; t, tibia; μCT, micro-computed tomography.

We next tested whether SOST downregulation was responsible for the bone phenotypes in the CKO mice. To this end, we took advantage of the *Dmp1-SOST* transgenic mouse that expresses the human *SOST* cDNA from a *Dmp1* regulatory sequence and therefore is expected to maintain SOST levels in osteocytes in the CKO mice. Previous characterization of the *Dmp1-SOST* mouse indicated that modest expression of SOST from the transgene reduced cancellous bone mass without affecting overall bone resorption.^24^ In our mating scheme, four relevant genotypes were produced at an equal Mendelian ratio of 1/4 (Figure 2a). We first imaged the littermate mice at P33 with X-ray, and found that the bones of the *Dmp1-SOST* mice were largely normal (SOST versus CTRL), but all CKO mice presented similar abnormal bone morphology regardless of *Dmp1-SOST* (SOST;CKO versus CKO; Figure 2b). In particular, the CKO and the SOST;CKO mice exhibited a smaller bone diameter at the proximal metaphysis of the femur (red arrow) and throughout the tibia when compared to the control (CTRL) or SOST littermates (Figure 2b). Imaging and quantification of the cortical bone with μCT confirmed that the overall bone size (Tt. Ar) was smaller at the proximal femur (red arrow) in the CKO and the SOST;CKO mice, but the bone area (Ct. Ar) was normal, resulting in a smaller medullary space (Ma. Ar) than CTRL or SOST (Figure 2c and d). Thus, forced expression of SOST does not rescue the cortical bone phenotype caused by Bmpr1a deletion.

**Figure 3:**
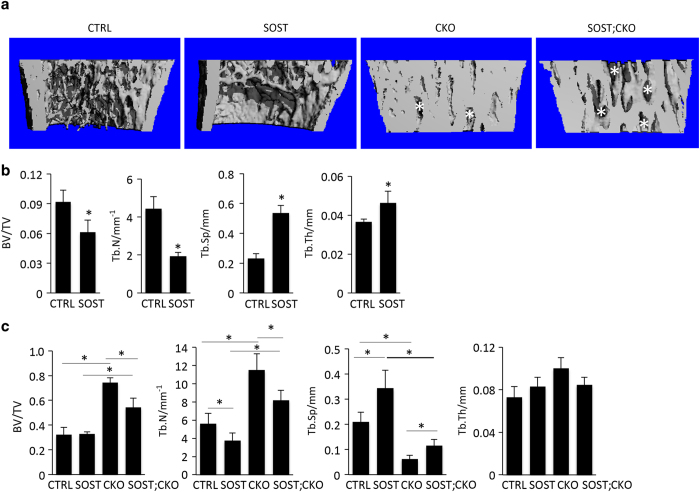
Forced expression of SOST reduces cancellous bone mass in Bmpr1a-deficient mice. (**a**) μCT 3D reconstruction images of the metaphyseal region of distal femur in littermate mice at P33. Asterisk denotes marrow space. (**b**) μCT quantification of cancellous bone in the distal metaphyseal region of the femur. *P<0.05, Student’s *t*-test. (**c**) μCT quantification of total metaphyseal bone (including both cancellous and cortical bone). Note that the parameters may not accurately reflect cancellous bone properties especially in CTRL and SOST mice due to the inclusion of both cancellous and cortical bone in the analysis. *P<0.001, one-way ANOVA, *n*=5 for CTRL and CKO, *n*=6 for SOST, *n*=7 for SOST;CKO. ANOVA, analysis of variance; CTRL, control; SOST, sclerostin; 3D, three dimensional; μCT, micro-computed tomography.

The X-ray images revealed that the cancellous bone region in the SOST;CKO mice was consistently shorter and less radiopaque than that in the CKO mice (Figure 2b, green line). We therefore examined the metaphyseal region of the femur in more detail with μCT. We confirmed that the SOST mice had considerably less cancellous bone than the CTRL littermate, as indicated by both 3D reconstruction images and quantification of the cancellous bone parameters (Figure 3a and b). The reconstruction images also revealed that both CKO and SOST;CKO mice possessed much more bone than either CTRL or SOST littermates, but the SOST;CKO mice exhibited considerably more marrow space within the cancellous bone region than the CKO mice (Figure 3a, asterisks). Due to the fact that cancellous versus cortical bone could not be reliably distinguished in the CKO and the SOST;CKO mice, we measured the total metaphyseal bone mass across all four genotypes. Such measurements detected no significant difference in BV/TV between CTRL and SOST mice, indicating that the difference in cancellous bone between the two was obscured by the inclusion of the cortical bone (Figure 3c). However, the SOST;CKO mice had significantly less metaphyseal bone mass (BV/TV) than the CKO littermate, although still more than CTRL or SOST (Figure 3c). The SOST transgene also significantly reduced trabecular number (Tb. N) and increased trabecular separation (Tb. Sp) in the CKO background. Overall, SOST expression partially corrects the phenotype of high cancellous bone mass caused by Bmpr1a deletion.

### Bmp signaling restricts preosteoblast proliferation partly through SOST induction

**Figure 4:**
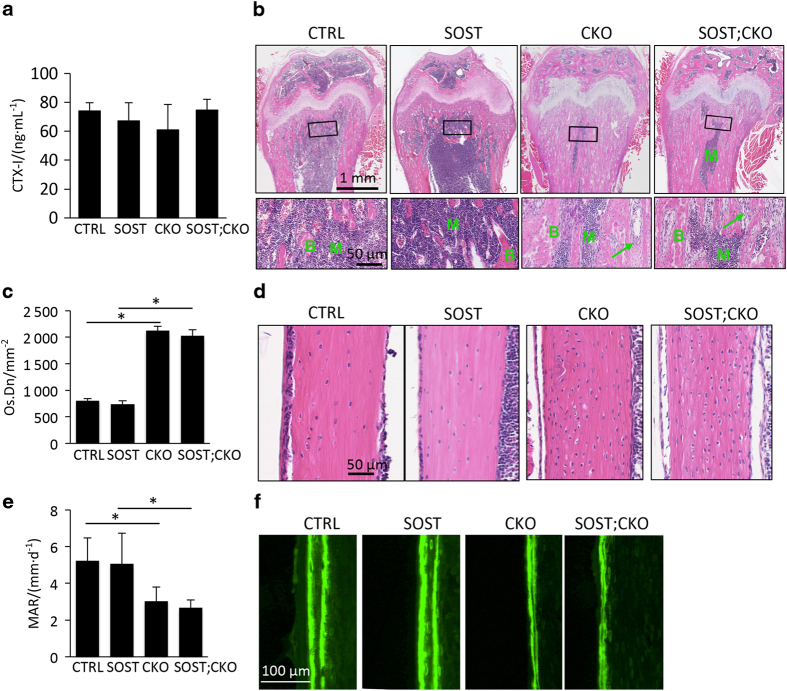
SOST expression partially rescues cancellous but not cortical bone phenotype caused by Bmpr1a deletion. (**a**) Serum CTX-I assays, *n*=3. (**b**) Representative images of H&E stained sections of the distal femur at P33. Boxed regions are shown at a higher magnification in lower panels. Arrow denotes accumulation of osteoblasts. (**c** and **d**) H&E staining (**c**) and quantification of osteocyte density (**d**) in cortical bone of the femur at P33. *P<0.001, two-way ANOVA, *n*=3. (**e**) Representative images of calcein double labeling at periosteal surface in P33 littermate mice. (**f**) Quantification of MAR. *P<0.001, owo-way ANOVA, *n*=3. ANOVA, analysis of variance; B, bone; M, marrow; SOST, sclerostin.

**Figure 5:**
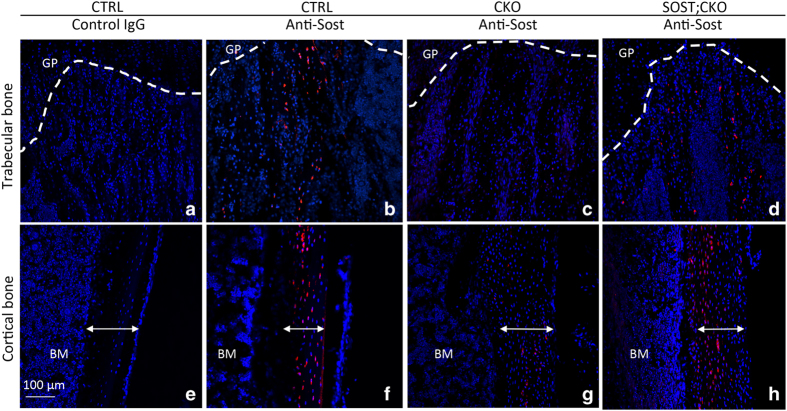
SOST expression is detected by immunofluorescence in both cancellous and cortical bone in SOST; CKO mice. Immunostaining was performed with non-immune IgG (**a** and **e**) or an antibody recognizing both murine and human sclerostin (**b**–**d** and **f**–**h**) on frozen sections of the femur. Blue, nuclear staining by DAPI; BM, bone marrow; double-headed arrow, cortical bone; GF, growth plate; red, antibody staining against both murine and human sclerostin; SOST, sclerostin.

We next investigated further the effect of SOST on the cancellous bone phenotype. Serum CTX-I assays detected no difference among all four genotypes, indicating that suppression of bone resorption was unlikely to be the main mechanism for the excessive bone mass caused by Bmpr1a deletion, or the partial rescue by SOST (Figure 4a). Histology of the femur confirmed the presence of more marrow space within the cancellous bone region of the SOST;CKO than the CKO mouse (Figure 4b, “M”). However, similar to the CKO littermates, the SOST;CKO mice showed an accumulation of osteoblasts between the neighboring trabeculae in areas devoid of bone marrow (Figure 4b, arrow). In addition, SOST overexpression did not alter the osteocyte density that was markedly increased by Bmpr1a deletion (Figure 4c and d, CKO vs SOST;CKO). Likewise, SOST did not modify the marked decrease in periosteal osteoblast activity as determined by calcein double labeling in the CKO background (Figure 4e and f, CKO vs SOST;CKO). Immunofluorescence staining with an antibody recognizing both murine and human sclerostin indicated that the protein was elevated in the osteocytes of both cancellous and cortical bone in the SOST;CKO over the CKO mice (Figure 5). It should be noted, however, that the overall level of sclerostin in the SOST;CKO mice was still lower than that in CTRL. This result is consistent with our previous characterization that the Dmp1-SOST transgene is expressed at a relatively low level. Nonetheless, these results demonstrate that forced expression of SOST was sufficient to reduce cancellous bone formation in the Bmpr1a-deficient mice.

**Figure 6:**
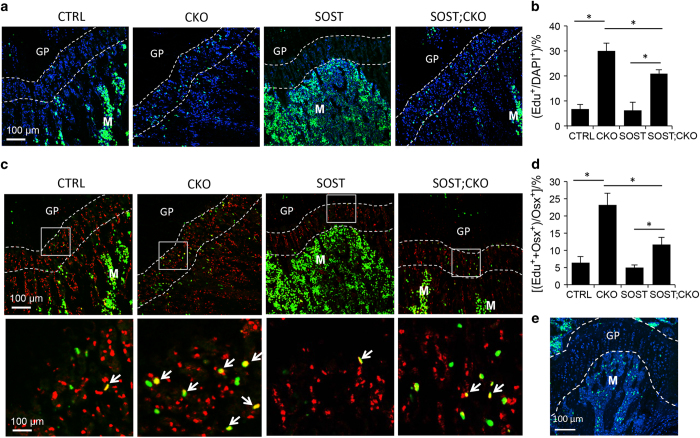
Forced expression of SOST partially corrects hyperproliferation caused by Bmpr1a deletion. (**a**) Representative images of distal femur labeled with EdU at P33. EdU signal is in green and DAPI nuclei staining in blue. (**b**) EdU labeling index over total cells in chondro-osseous junction. (**c**) Representative images of Osx immunofluorescence staining and EdU labeling at P33. Boxed regions in chondro-osseous junction below growth plate are shown at a higher magnification in lower panels. EdU is in green and Osx in red; Arrows denote double positive cells. (**d**) EdU labeling index among Osx+ preosteoblasts in chondro-osseous junction. (**e**) Negative control for Osx immunofluorescence staining. A non-immuno IgG detected no red signal on section from CTRL mouse. Region between dotted lines denotes chondro-osseous junction chosen for quantification [100 μm region immediately under growth plate (GP)]. GP: growth plate; M: marrow. *P<0.001, two-way ANOVA, *n*=3. ANOVA, analysis of variance; CTRL, control; SOST, sclerostin.

As we have previously shown that Bmpr1a deletion stimulates proliferation of preosteoblasts to increase cancellous bone formation, we next examined the effect of SOST expression on cell proliferation with the EdU labeling technique. The method detected a relatively low proliferation index (~5% EdU^+^) among all cells within the chondro-osseous junction in both wild-type and SOST mice (Figure 6a and b, CTRL vs SOST). Double labeling with an Osx antibody showed that the Osx^+^ preosteoblasts also proliferated at relatively low rate in either wild-type or SOST mice (Figure 6c and d, CTRL, SOST). However, Bmpr1a deletion markedly increased the proliferation index among either all cell or the Osx^+^ preosteoblasts at the chondro-osseous junction (Figure 6b and d, CKO vs CTRL). Importantly, the increased proliferation caused by Bmpr1a deletion was notably reduced by SOST overexpression even though the labeling index remained significantly higher than that in the CTRL or SOST mice (Figure 6b and d). Statistical analyses with two-way analysis of variance indicated a significant effect of SOST overexpression on Bmp1a deletion (interaction *P*-value <0.001). We confirmed the specificity of the Osx antibody, as a non-immune IgG did not detect any positive cells (Figure 6e). Thus, forced expression of SOST partially suppresses hyperproliferation of osteoblast precursors caused by Bmpr1a deletion.

### High bone mass allele of Lrp5 does not rescue periosteal bone growth in Bmpr1a-deficient mice

**Figure 7:**
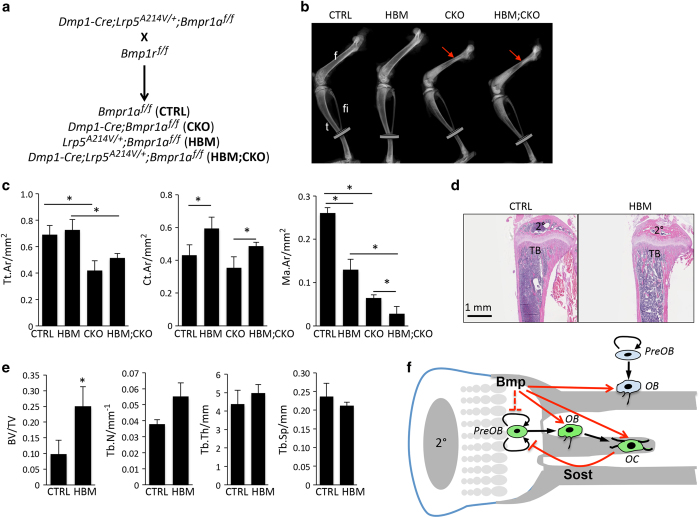
High bone mass Lrp5 mutant allele does not restore periosteal bone growth in Bmpr1a-deficient mice. (**a**) Mating scheme. (**b**) Representative X-ray images of hindlimbs of littermate mice at P33. f: femur; fi: fibula; t: tibia. Arrows denote smaller diameter in the proximal femur specific to CKO and HBM;CKO mice. Discs mark region of tibia (tibia-fibula junction) analyzed by μCT in (**c**). (**c**) Quantification of cortical bone parameters by μCT at region of tibia marked by disc in B. *P<0.01, two-way ANOVA, *n*=4. (**d**) Representative images for H&E staining of longitudinal sections through the proximal tibia. Note more bone in trabecular region (TB) and secondary ossification center (2°) in HBM than CTRL. (**e**) Quantification of trabecular bone parameters by μCT. (**f**) Model for Bmp signaling in osteoblast lineage cells. Bmp signaling via Bmpr1a directly promotes osteoblast activity in both trabecular and periosteal bone. Bmp also acts on osteocytes to induce SOST that in turn suppresses preosteoblast proliferation in trabecular bone region. On the other hand, production of periosteal osteoblasts is not altered by increased Wnt signaling. Trabecular versus periosteal osteoblast lineage is depicted in green versus blue. Red arrow and blocked arrow indicate stimulation and inhibition, respectively. Dashed line indicates potential action. ANOVA, analysis of variance; CTRL, control; OB, osteoblast; OC, osteocyte; PreOB, preosteoblast; 2°, secondary ossification center; μCT, micro-computed tomography.

The data so far indicate that SOST downregulation does not contribute to the periosteal growth defect caused by Bmpr1a deletion. This result is expected as SOST generally suppresses bone formation through inhibition of Wnt signaling. We next tested whether hyperactivation of Wnt signaling could overcome the deficit in periosteal growth. To this end, we utilized the *Lrp5^A214V/+^* knock-in mouse that expresses from the endogenous Lrp5 locus a mutant Lrp5 allele (A214V) that is known to increase Wnt signaling and cause high bone mass (HBM) in humans and mice. We generated littermate animals with or without the HBM Lrp5 allele expressed in either wild-type or Bmpr1a-deficient background (Figure 7a). X-ray imaging at P33 detected an increase in the cortical thickness of the long bones in the HBM mice over the control littermates (Figure 7b, CTRL vs HBM). However, expression of the HBM allele did not rescue the cross-sectional size of the cortical bone in the Bmpr1a mutant mice (Figure 7b, CKO vs HBM; CKO, red arrows). Quantitation of the tibial cortical bone at the tibia-fibula junction with μCT confirmed that the cross-sectional size (Tt. Ar) was similarly reduced by Bmpr1a deletion regardless of the HBM allele (Figure 7c, left). However, the HBM allele increased the cortical thickness (Ct. Ar) in both control and Bmpr1a-deficient mice, resulting similar decreases in the marrow space (Ma. Ar) (Figure 7c, middle and right). Therefore, although hyperactivation of Wnt signaling promotes endosteal bone formation, it does not rescue periosteal bone growth in the absence of Bmpr1a. To establish further the efficacy of the Lrp5 HBM allele in our experimental setting, we analyzed the trabecular bone phenotype in the HBM versus control mice. Histology showed a clear increase in trabecular bone mass in both primary and secondary ossification centers of the tibia (Figure 7d). Quantitative analyses of the proximal tibia with μCT revealed a twofold increase in trabecular bone mass (BV/TV) in HBM over control mice (Figure 7e). Overall, based on this and our previous study, we propose a model wherein Bmp signaling regulates cancellous bone formation by both enhancing osteoblast activity and restricting preosteoblast proliferation.^10^ The proliferation constrain is at least partly mediated by the induction of SOST in osteocytes but may also involve direct Bmp action on the preosteoblasts (Figure 7f). The proposition of direct inhibition of preosteoblast proliferation by Bmp is based on the fact that hyperproliferation was not fully corrected by the forced expression of SOST, but we cannot rule out that the lack of a full rescue might be due to the relatively low expression of SOST as previously documented.^28^ We further propose that Bmp promotes periosteal bone growth mainly through direct stimulation of osteoblast activity largely independent of Wnt signaling (Figure 7f).

## Discussion

We have investigated the role of Wnt signaling in mediating Bmpr1a function in bone. Specifically, we tested whether genetic manipulation of Wnt signaling could modify the bone phenotypes caused by Bmpr1a deletion, namely excessive accrual of cancellous bone and impaired periosteal growth of cortical bone. Whereas forced expression of SOST partially rescued the cancellous bone mass, a hyperactive form of Lrp5 did not ameliorate the defect in periosteal bone growth in the Bmpr1a-deficient background. Mechanistically, SOST alleviated the hyperproliferation of cancellous preosteoblasts caused by Bmpr1a deletion. These results demonstrate that Bmp signaling regulates bone formation through both Wnt-dependent and -independent mechanisms.

It is worth noting that Bmp signaling appears to exert different effects on endosteal versus periosteal bone growth. Although the deletion of Bmpr1a notably restricted periosteal bone growth throughout the tibia, it did not reduce the total amount of cortical bone. In calcein labeling experiments, we frequently observed double-labeled surfaces at the endosteum of the diaphysis in the CKO but not the wild-type mice at P33, indicating an increase of active osteoblasts over the quiescent lining cells on the endosteal bone surface in the absence of Bmpr1a. The reasons for the increase in active endosteal osteoblasts however, are currently unclear. Aside from the potential direct effects of Bmpr1a deletion, we suspect that an increase in mechanical stress due to the reduced cross-sectional bone size may prolong the productive life span of endosteal osteoblasts. The mechanical stress response model is appealing as it helps to explain the normal, but not excessive cortical bone mass in the CKO mice; this is an intriguing distinction from the HBM mice that possess an abnormally high amount of cortical bone mass even though the overall sectional size of the bone is normal at P33 (Figure 7c). Regardless of the exact mechanism, hyperactive Wnt signaling by the mutant Lrp5 stimulated excessive endosteal bone formation regardless of Bmpr1a. Thus, whereas Bmpr1a is epistatic to Wnt in stimulating periosteal bone growth, the opposite appears to be true in regards to endosteal bone formation.

The study has also revealed different responses by cancellous versus cortical bone to perturbation of Wnt signaling. Expression of SOST or the mutant Lrp5 reduced or increased cancellous bone mass, respectively, demonstrating a stimulatory effect of Wnt signaling in the cancellous bone compartment. In contrast, neither manipulation had any effect on the cross-sectional size of the long bones by postnatal 33 days, indicating the relative independence of periosteal bone growth on the level of Wnt signaling. As others have reported that the same mutant Lrp5 (A214V) leads to bigger bone sizes in 4-month-old mice, bone expansion at the periosteum may be more sensitive to hyperactive Wnt signaling in adults than in young animals.^29^ Recently, deletion of Sfrp4, a secreted antagonist of Wnt proteins, was shown to increase cancellous bone volume but reduce cortical thickness while expanding the cross-sectional size, perhaps though compartment-specific regulation of Bmp signaling.^30^ Further studies are warranted to elucidate fully the molecular basis for site-specific effects of Wnt perturbation on bone resorption and formation.

We have focused our study on the regulation of bone formation by Bmp signaling. Others have reported a similar increase in cancellous bone mass following deletion of *Bmpr1a* with *Col1-Cre^ER^* or *Dmp1-Cre* but attributed the phenotype mostly to the suppression of bone resorption.^19,20,21^ We, however, have not detected a significant difference in CTX-I levels between wild-type and *Dmp1-Cre*; *Bmpr1a^f/f^* (CKO) littermates at P33 (ref. 10) (this study). We are mindful, however, that the sample size in both studies was limited (*n*=3) and analyses of more mice might reveal differences between the genotypes. On the other hand, it is possible that the status of bone resorption in the mutant mice changes with age, as others noted a decrease of serum CTX-I levels in the CKO mice at 16 weeks of age.^21^ Of note, deletion of *Bmpr1a* or *Smad4* in mature osteoblasts (*Og2-Cre*) also led to a decrease in bone resorption most notable in the aged mice.^17,31^ Thus, whereas increased osteoblast number appears to drive the excessive cancellous bone mass early in life in the CKO mice, a decrease in bone resorption could exacerbate the phenotype in aged mice.

## References

[bib1] Urist MR, Mikulski A, Lietze A. Solubilized and insolubilized bone morphogenetic protein. Proc Natl Acad Sci USA 1979; 76: 1828–1832. 221908 10.1073/pnas.76.4.1828 PMC383485

[bib2] Salazar VS, Gamer LW, Rosen V. BMP signalling in skeletal development, disease and repair. Nat Rev Endocrinol 2016; 12: 203–221. 26893264 10.1038/nrendo.2016.12

[bib3] Wu MY, Hill CS. Tgf-beta superfamily signaling in embryonic development and homeostasis. Dev Cell 2009; 16: 329–343. 19289080 10.1016/j.devcel.2009.02.012

[bib4] Miyazono K, Kamiya Y, Morikawa M. Bone morphogenetic protein receptors and signal transduction. J Biochem 2010; 147: 35–51. 19762341 10.1093/jb/mvp148

[bib5] Wrana JL, Attisano L, Wieser R et al. Mechanism of activation of the TGF-beta receptor. Nature 1994; 370: 341–347. 8047140 10.1038/370341a0

[bib6] Massague J. TGFbeta signalling in context. Nat Rev Mol Cell Biol 2012; 13: 616–630. 22992590 10.1038/nrm3434 PMC4027049

[bib7] Wharton K, Derynck R. TGFbeta family signaling: novel insights in development and disease. Development 2009; 136: 3691–3697. 19855012 10.1242/dev.040584

[bib8] Ghosh-Choudhury N, Mandal CC, Das F et al. c-Abl-dependent molecular circuitry involving Smad5 and phosphatidylinositol 3-kinase regulates bone morphogenetic protein-2-induced osteogenesis. J Biol Chem 2013; 288: 24503–24517. 23821550 10.1074/jbc.M113.455733 PMC3750149

[bib9] Ghosh-Choudhury N, Abboud SL, Nishimura R et al. Requirement of BMP-2-induced phosphatidylinositol 3-kinase and Akt serine/threonine kinase in osteoblast differentiation and Smad-dependent BMP-2 gene transcription. J Biol Chem 2002; 277: 33361–33368. 12084724 10.1074/jbc.M205053200

[bib10] Lim J, Shi Y, Karner CM et al. Dual function of Bmpr1a signaling in restricting preosteoblast proliferation and stimulating osteoblast activity in mouse. Development 2016; 143: 339–347. 26657771 10.1242/dev.126227 PMC4725340

[bib11] Benazet JD, Pignatti E, Nugent A et al. Smad4 is required to induce digit ray primordia and to initiate the aggregation and differentiation of chondrogenic progenitors in mouse limb buds. Development 2012; 139: 4250–4260. 23034633 10.1242/dev.084822

[bib12] Lim J, Tu X, Choi K et al. BMP-Smad4 signaling is required for precartilaginous mesenchymal condensation independent of Sox9 in the mouse. Dev Biol 2015; 400: 132–138. 25641697 10.1016/j.ydbio.2015.01.022 PMC4361319

[bib13] Bandyopadhyay A, Tsuji K, Cox K et al. Genetic analysis of the roles of BMP2, BMP4, and BMP7 in limb patterning and skeletogenesis. PLoS Genet 2006; 2: e216. 17194222 10.1371/journal.pgen.0020216 PMC1713256

[bib14] Retting KN, Song B, Yoon BS et al. BMP canonical Smad signaling through Smad1 and Smad5 is required for endochondral bone formation. Development 2009; 136: 1093–1104. 19224984 10.1242/dev.029926 PMC2668702

[bib15] Yoon BS, Ovchinnikov DA, Yoshii I et al. Bmpr1a and Bmpr1b have overlapping functions and are essential for chondrogenesis in vivo. Proc Natl Acad Sci USA 2005; 102: 5062–5067. 15781876 10.1073/pnas.0500031102 PMC555995

[bib16] Tsuji K, Bandyopadhyay A, Harfe BD et al. BMP2 activity, although dispensable for bone formation, is required for the initiation of fracture healing. Nat Genet 2006; 38: 1424–1429. 17099713 10.1038/ng1916

[bib17] Mishina Y, Starbuck MW, Gentile MA et al. Bone morphogenetic protein type IA receptor signaling regulates postnatal osteoblast function and bone remodeling. J Biol Chem 2004; 279: 27560–27566. 15090551 10.1074/jbc.M404222200

[bib18] Tan XH, Weng TJ, Zhang JH et al. Smad4 is required for maintaining normal murine postnatal bone homeostasis. J Cell Sci 2007; 120: 2162–2170. 17550966 10.1242/jcs.03466 PMC2692485

[bib19] Kamiya N, Ye L, Kobayashi T et al. BMP signaling negatively regulates bone mass through sclerostin by inhibiting the canonical Wnt pathway. Development 2008; 135: 3801–3811. 18927151 10.1242/dev.025825 PMC2694443

[bib20] Kamiya N, Ye L, Kobayashi T et al. Disruption of BMP signaling in osteoblasts through type IA receptor (BMPRIA) increases bone mass. J Bone Miner Res 2008; 23: 2007–2017. 18684091 10.1359/JBMR.080809 PMC2686924

[bib21] Kamiya N, Shuxian L, Yamaguchi R et al. Targeted disruption of BMP signaling through type IA receptor (BMPR1A) in osteocyte suppresses SOST and RANKL, leading to dramatic increase in bone mass, bone mineral density and mechanical strength. Bone 2016; 91: 53–63. 27402532 10.1016/j.bone.2016.07.002

[bib22] Lu Y, Xie Y, Zhang S et al. DMP1-targeted Cre expression in odontoblasts and osteocytes. J Dent Res 2007; 86: 320–325. 17384025 10.1177/154405910708600404

[bib23] Mishina Y, Hanks MC, Miura S et al. Generation of Bmpr/Alk3 conditional knockout mice. Genesis 2002; 32: 69–72. 11857780 10.1002/gene.10038

[bib24] Rhee Y, Allen MR, Condon K et al. PTH receptor signaling in osteocytes governs periosteal bone formation and intracortical remodeling. J Bone Miner Res 2011; 26: 1035–1046. 21140374 10.1002/jbmr.304 PMC3179307

[bib25] Cui Y, Niziolek PJ, Macdonald BT et al. Lrp5 functions in bone to regulate bone mass. Nat Med 2011; 17: 684–691. 21602802 10.1038/nm.2388 PMC3113461

[bib26] Bouxsein ML, Boyd SK, Christiansen BA et al. Guidelines for assessment of bone microstructure in rodents using micro-computed tomography. J Bone Miner Res 2010; 25: 1468–1486. 20533309 10.1002/jbmr.141

[bib27] Karner CM, Esen E, Chen J et al. Wnt protein signaling reduces nuclear acetyl-CoA levels to suppress gene expression during osteoblast differentiation. J Biol Chem 2016; 291: 13028–13039. 27129247 10.1074/jbc.M115.708578 PMC4933220

[bib28] Tu X, Rhee Y, Condon KW et al. Sost downregulation and local Wnt signaling are required for the osteogenic response to mechanical loading. Bone 2012; 50: 209–217. 22075208 10.1016/j.bone.2011.10.025 PMC3246572

[bib29] Niziolek PJ, Farmer TL, Cui Y et al. High-bone-mass-producing mutations in the Wnt signaling pathway result in distinct skeletal phenotypes. Bone 2011; 49: 1010–1019. 21855668 10.1016/j.bone.2011.07.034 PMC3412139

[bib30] Simsek Kiper PO, Saito H, Gori F et al. Cortical-Bone Fragility--Insights from sFRP4 Deficiency in Pyle's Disease. N Engl J Med 2016; 374: 2553–2562. 27355534 10.1056/NEJMoa1509342 PMC5070790

[bib31] Tan X, Weng T, Zhang J et al. Smad4 is required for maintaining normal murine postnatal bone homeostasis. J Cell Sci 2007; 120 (Pt 13): 2162–2170. 17550966 10.1242/jcs.03466 PMC2692485

